# *Trans*-ε-Viniferin Encapsulation in Multi-Lamellar Liposomes: Consequences on Pharmacokinetic Parameters, Biodistribution and Glucuronide Formation in Rats

**DOI:** 10.3390/nu13124212

**Published:** 2021-11-24

**Authors:** Pauline Beaumont, Chrystel Faure, Arnaud Courtois, Michael Jourdes, Axel Marchal, Pierre-Louis Teissedre, Tristan Richard, Claude Atgié, Stéphanie Krisa

**Affiliations:** 1 INRAE, Bordeaux INP, UR OENOLOGIE, EA 4577, USC 1366, ISVV, Université de Bordeaux, 33140 Villenave d’Ornon, France; pauline.beaumont@u-bordeaux.fr (P.B.); arnaud.courtois@u-bordeaux.fr (A.C.); michael.jourdes@u-bordeaux.fr (M.J.); axel.marchal@u-bordeaux.fr (A.M.); pierre-louis.teissedre@u-bordeaux.fr (P.-L.T.); tristan.richard@u-bordeaux.fr (T.R.); 2 CNRS, Bordeaux INP, CBMN, UMR 5248, Université de Bordeaux, 33600 Pessac, France; chrystel.faure@enscbp.fr; 3 Centre Antipoison et de Toxicovigilance de Nouvelle Aquitaine, Bâtiment UNDR, CHU de Bordeaux, Place Amélie Raba Léon, 33076 Bordeaux, France

**Keywords:** *Trans*-ε-viniferin, resveratrol dimer, pharmacokinetic, liposome encapsulation, biodistribution

## Abstract

*Trans*-ε-viniferin (εVin) is a resveratrol dimer exhibiting promising biological activities for human health. Its bioavailability being low, the development of encapsulation methods would be used to overcome this issue. The aim of this study was to measure the consequences of the encapsulation of εVin in multilamellar liposomes on its pharmacokinetic parameters, metabolism and tissue distribution in rats. After oral administration of εVin (20 mg/kg body weight), either as free or encapsulated forms, plasmas were sequentially collected (from 0 to 4 h) as well as liver, kidneys and adipose tissues (4 h after administration) and analyzed by LC-HRMS. The glucuronide metabolites (εVG) were also produced by hemisynthesis for their quantification in plasma and tissues. The encapsulation process did not significantly modify the pharmacokinetic parameters of εVin itself. However, a significant increase of the T_1/2_ was noticed for εVG after administration of the encapsulated form as compared to the free form. An accumulation of εVin and εVG in adipose tissues was noticed, and interestingly a significant increase of the latter in the mesenteric one after administration of the encapsulated form was highlighted. Since adipose tissues could represent storage depots, and encapsulation allows for prolonging the exposure time of glucuronide metabolites in the organism, this could be of interest to promote their potential biological activities.

## 1. Introduction

*Trans*-ε-viniferin (εVin) is a resveratrol dehydrodimer belonging to the stilbene’s family. Stilbenes are phytoalexins present in several edible plant families [1]. The main source of these compounds in European food is grapes [2] and their derivative products such as grapes juice or wine, but also peanuts and red fruits [3,4]. The average daily consumption of stilbenes differs according to several criteria but is estimated at 0.5 mg/person/day according to a Spanish study, and 0.3 mg/person/day according to a Chinese one [3,5,6].

Although resveratrol is the most widely studied stilbene for its biological activities, recent research focuses on the biological properties of other stilbenes. Indeed, numerous studies on the biological properties of εVin, a dimer present in red wines [7] in the wood of Vitis vinifera (up to 7.7 g/kg dry weight, more abundant than resveratrol [8]), have shown that this molecule could have some benefits for human health.

εVin has indeed shown antioxidant activities, by inhibiting the radical ABTS^•+^ [9,10] and also by inhibiting peroxidation of β-carotene, or by limiting oxidation of DMSO/O_2_- system [11]. εVin also reduced nitric oxide production by microglia and macrophages (BV-2 cells and RAW 264.7 cells) activated by lipopolysaccharide, suggesting some anti-inflammatory properties [12,13]. Moreover, studies have shown interesting εVin neuroprotective activities, by inhibiting Aβ aggregation involved in Alzheimer’s disease [14,15], or by activating sirtuin 3 and protecting mitochondrial functions in a huntingtin mutant cell line [16]. Experiments on cancer cell lines like those derived from hepatocytes, fibroblasts, and leukemia cells or lymphoids revealed antiproliferative activities of εVin [17,18,19,20,21,22], even if other studies suggested no or very low activity [23,24]. Other research groups have shown more efficient in vitro anti-hepatitis C virus [25], antidepressant [26], and cardioprotective activities by decreasing the systolic blood pressure in spontaneously hypertensive rats by comparing to its monomer resveratrol [27,28]. Moreover, εVin is also promising for its anti-adipogenic properties insofar as it has been shown that this compound decreased lipid accumulation and adipogenesis marker gene expression such as Peroxisome Proliferator-activated Receptor gamma (PPARϒ) in a 3T3-L1 adipocytes cell line [29]. The decrease in body weight gain in rat fed with a high fat diet and the reduction of the mass of white adipose tissues in mice after oral administration of εVin [29,30] highlight its potential in vivo biological activities. These interesting properties lead to asking the question of its fate in the systemic circulation after ingestion.

Nowadays, and to our knowledge, the only study about εVin pharmacokinetic characterization and bioavailability after oral administration was published by Kim et al. [31]. They showed a εVin maximal concentration (C_max_) of 42 ng/mL for a maximal time (T_max_, time to reach the C_max_) between 15 and 30 min after oral administration of 40 mg/kg body weight (bw) in mice, and calculated a bioavailability of 0.77% [31]. This very poor bioavailability could be explained by a low absorption through the intestinal barrier [32] and/or a strong intestinal and/or liver metabolism. It is now well documented that stilbenes are indeed subjected to biotransformations such as sulfation and glucuronidation, via sulfotransferase and UDP-glucuronosyltransferase, respectively. These detoxification processes take place mainly in the liver but also to a significant level in the enterocytes of the small intestine. These enzymatic transformations result in an increase of the compound hydrophilicity for an easier elimination into bile and urine. Two recent studies from our laboratory clearly confirmed a high metabolism of εVin in vitro (S9 liver extract) and in vivo (in rats). In this last in vivo study, the pharmacokinetic parameters calculated, after intraperitoneal injection in rats, a C_max_ at 3.09, 5.16 and 0.2 µg/mL for εVin, its glucuronide (εVG) and its sulfate (εVS) metabolites, respectively, for an equivalent T_max_ at 1 h [33]. However, it is noteworthy that, in these previous studies, the concentrations of these metabolites were expressed as the equivalent of the native form, as εVG and εVS are not commercially available. Metabolism was also observed for an isomer of εVin, δ-viniferin, after an oral administration at 70 mg/kg bw in rats. Indeed, Mao et al. quantified the glucuronides of δ-viniferin, after hydrolysis with glucuronidase, and measured a concentration of glucuronide metabolites up to 7-fold higher than the native form [34]. Finally, the quantification of εVin and its metabolites in adipose tissue after intraperitoneal administration showed an accumulation of the molecule mainly in its native form, suggesting a storage role for this tissue [33].

Due to the very poor bioavailability of stilbenes, developing innovative formulations like micro-encapsulation could be a solution to improve their bioavailability [35]. Several methods have been proposed to increase the stability, solubility and bioavailability of polyphenols [36,37,38] such as single and double emulsions, co-crystallization, encapsulation in yeasts and liposomes. Some studies on polyphenols encapsulation (such as resveratrol, curcumin and quercetin) have shown a real efficiency in improving their pharmacokinetic parameters and their bioavailability [39], by increasing C_max_ or T_1/2_ [40,41,42,43].

Liposomes are good candidates for in vivo applications because of their biocompatibility and low toxicity. The lipid bilayers that constitute liposomes allow for protecting polyphenols against UV irradiation and *cis*-*trans* isomerization [44,45], increasing their stability and preserving their anti-oxidant properties [46]. Among the different types of liposome encapsulation, multi-lamellar liposomes (MLLs) are advantageous for the encapsulation of polyphenols because of the presence of several bilayers in which lipophilic molecules, such as εVin, are inserted [47], leading to a higher encapsulation rate. Onion-type MLLs, also called spherulites©, are made up of lipid bilayers up to the core of vesicles, contrary to classical MLLs that display a limited number of bilayers and a large water core. Recently, we produced εVin-loaded onions displaying a high encapsulation rate [48], considerably increasing its water solubility, and showing a decreased cytotoxicity of encapsulated εVin on Caco-2 cell line [45].

This study aimed to investigate the consequences of εVin encapsulation in onion-type MLLs on its pharmacokinetic parameters and tissular distribution after oral administration in rats. Plasma and tissue concentrations of the native form (εVin) will be assessed, as well as those of glucuronides, through hemisynthetic production of these metabolites.

## 2. Materials and Methods

### 2.1. Chemical and Stock Solutions

#### 2.1.1. Chemical and Reagents

εVin was produced as previously described [49], by extraction from grape shoot powder batch, and purity was confirmed by ^1^H NMR (>89%). P75 (soya bean lecithin with 68–74% phosphatidylcholine and 7–11% phosphatidylethanolamine) was purchased from Lipoid GmbH (Ludwigshafen, Germany). Tween 80, K_2_CO_3_, acetobromo-glucuronic acid methyl-ester, pyridine chlorosulfonic acid and propylene glycol were purchased from Sigma-Aldrich (Saint Quentin Fallavier, France). Methanol, ethanol and acetonitrile (LC-MS)-grade, trifluoroacetic acid (TFA) and formic acid were purchased from Fisher Scientific (Loughborough, UK), and ultra-pure water was produced using Purelab Ultra System (Elga Lab Water, High Wycombe, UK).

#### 2.1.2. Metabolites Production

**Figure 1:**
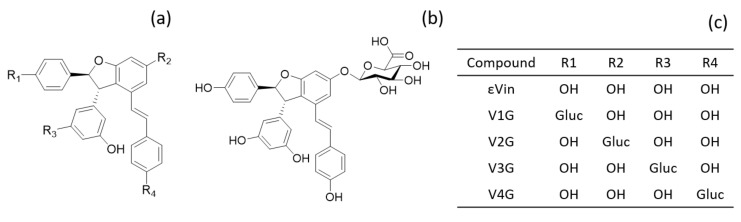
Structure of the 4 isomers of εVin. (**a**) basic structure; (**b**) structure of V2G; (**c**) location of glucuronide group (gluc) depending on the isomer.

The four glucuronides (Figure 1) were obtained by hemisynthesis as previously described [50] with some modifications. One molar equivalent εVin was dissolved in ethanol and then mixed with 20 molar equivalents of K_2_CO_3_. Six molar equivalents of acetobromo-glucuronic acid methyl-ester dissolved in ethanol were added, and the reaction mixture was stirred at 50 °C for 24 h under agitation. The reaction was then stopped by lowering the pH below 5 with 1% formic acid. Solvents were evaporated with a vacuum rotatory evaporator before redissolving sample in methanol:water (50:50, *v*:*v*). The mixture was injected into semi-preparative high-performance liquid chromatography equipped with a binary pump and UV-VIS detector used at 320 nm (Prostar 325, Varian, Palo Alto, CA, USA). Solvents A and B were ultra-pure water and acetonitrile:water (50:50, *v*:*v*), respectively, both acidified with 0.025% of TFA. The gradient, used with a 2 mL/min flow rate, was as follows: 0 min, 38% B; 0–55 min, 38–48% B; 55–57 min, 48–100% B; 57–60 min, 100% B; 60–62 min, 100–38% B; 62–64 min; 38% B. Metabolites were individually collected and then injected in UPLC-DAD-MS to ensure their purity before being freeze-dried. For method validation and a calibration curve, V2G (Figure 1) was used as it is the most predominant isomer found in rats [33].

#### 2.1.3. Stock Solutions and Calibration Curves

εVin and V2G were precisely weighed and dissolved in ultra-pure (HPLC)-grade methanol to obtain a 1 mg/mL concentration. Stock solutions were aliquoted and stored at −20 °C until use. For calibration curves, dilutions were applied to give the following concentrations in methanol:water (50:50, *v*:*v*): 0.5, 1, 2.5, 5, 10, 25, 50, 100 and 250 ng/mL and 25, 50, 100, 250, 500, 1000, 2500 ng/mL for εVin and V2G, respectively.

### 2.2. εVin-Loaded MLLs

#### 2.2.1. Preparation of Onions-Type MLLs

MLLs were produced as previously described [33,48]. Briefly, P75, Tween 80 and εVin (52.5 wt%, 11.5 wt% and 4 wt% of the final composition, respectively) were first individually solubilized in ethanol before being mixed together. The organic solvent was then removed by evaporation under N_2_ flux (0.1 bar). The mixture was freeze-dried (48 h) and precisely weighed. The first half of ultra-pure water required (16 wt%) was added. Shearing using a microspatula was performed (1 min) before adding the other half of water (16 wt%) and starting three cycles of shear stress. One shear stress cycle consisted of 5 min of shearing with the spatula and 5 min of centrifugation (2000 rpm). The resulting viscous phase, made of MLLs in close contact, was kept at 4 °C for 24 h, and three additional shear stress cycles were then operated.

In Vivo experiments on rats were performed using encapsulated εVin. To remove un-encapsulated εVin, MLLs were suspended in ultra-pure water (20 mg/mL, 40 mL per tube). An initial centrifugation (10 min, 500 rpm) allowed the precipitation of potential εVin aggregates. The supernatants were then ultra-centrifuged for three hours at 100,000× *g* and 8 °C (fast acceleration, slow deceleration), and supernatants were removed to keep only εVin encapsulated in MLLs.

#### 2.2.2. Characterization

The multi-lamellar character of the liposomes was confirmed by polarized light microscopy (Olympus BX51, ×60 and ×100 magnifications, Hamburg, Germany). The encapsulation rate of εVin in MLLs was determined using UV-Vis spectrophotometry and using an adsorption filtration method as previously developed [48]. The liposomes distribution size was established using dynamic light scattering (DLS, Vasco particle size analyzer, Cordouan Technologies Ltd., Pessac, France) with a refractive index of 1.33 and 1.45 for water and liposomes, respectively. The measurement was done in triplicate and results were analyzed using the Cumulant method. Finally, the zeta potential was determined using ZetaSizer Nano Series (Malvern Instrument Ltd., Worcestershire, UK).

### 2.3. Pharmacokinetic Studies

#### 2.3.1. Animals and Treatments

Experiments were conducted in accordance with Directive 2010/63/EU [51] and approved by the Bordeaux University Institutional Ethics Committee for Animal Research (IEC-AR CEEA 50, MESR approval A18380). Male Wistar rats (Janvier-LABS, Saint Berthevin, France) were maintained in controlled conditions: 12 h light/12 h dark cycle, humidity 50–60% and an ambient temperature of 24 ± 1 °C (animal facility agreement number: B33-063-917). Free access to food and water was allowed up to 16 h before experiments started. Rats were randomly divided into two groups. Group A (*n* = 5, average weight = 307.8 ± 7.7 g) received εVin dissolved in propylene glycol (5.25 mg/mL), while group B (*n* = 6, average weight = 308 ± 10.7 g) received the same dose of εVin but encapsulated in MLLs and suspended in ultra-pure water. Both solutions were administrated by oral gavage and precisely measured to reach a dose of 20 mg/kg bw of εVin. Blood was collected through the tail vein, making a slanting incision during the first collection. Rats were slightly anesthetized by isoflurane before every blood sample collection (600 µL) at the following time points: 20, 40, 60, 100, 140, 180 and 210 min after gavage. They were sacrificed at 240 min by a high dose of isoflurane and rapid exsanguination with saline solution injected in the right ventricular cardiac cavity with a peristaltic pump (10 mL/min). All blood samples were collected in heparinized tubes and centrifuged (10,000× *g*, 10 min) before sampling plasma. The liver, kidneys and four white adipose tissues (epididymal, retroperitoneal, mesenteric and subcutaneous) were removed, weighed and immediately immersed in liquid nitrogen. All blood and tissues samples were kept at −80 °C until analysis.

#### 2.3.2. Extraction of εVin and Metabolites from Plasma and Tissues

Extractions were performed as previously described [33] with some modifications to improve quantification. For plasma extraction, 360 µL of methanol (4 °C) were added to an aliquot of 120 µL of plasma. The sample was vortexed during three minutes before centrifugation (30 min, 12,000× *g*, 4 °C). The supernatant (380 µL) was removed and evaporated using a SpeedVac concentrator (Labconco CentriVap, Thermo Fisher Scientific, Waltham, MA, USA). Residue was kept in −80 °C before analysis. For tissues extraction, tissues (about 0.5 g) were first cut with scalpels before being mixed with 4 mL of methanol:water (80:20, *v*:*v*). Samples were homogenized with an Ultra-Turrax homogenizer (IKA, Staufen, Deutschland) before being intensely vortexed. Then, samples were centrifuged 20 min at 10,000× *g* and 4 °C before sampling 3 mL of supernatant. All supernatants were evaporated using a SpeedVac concentrator and kept at −80 °C. Residues were reconstituted in 60 µL and 250 µL of methanol:water (50:50, *v*:*v*) or plasma and tissue, respectively. They were vortexed intensely and centrifuged at 12,000× *g* during 30 min at 4 °C before being injected the supernatant in LC-HRMS.

### 2.4. LC-HRMS Quantification

The quantification of εVin and the total of glucuronides (εVG) in plasma and tissues was performed as previously described [33] with some modifications concerning the mass parameters. The U-HPLC separation was carried out with a Vanquish Flex system (Thermo Fisher Scientific, Les Ulis, France) consisting in a binary pump, an autosampler and a heated column compartment. The liquid chromatography separation was performed as previously detailed using C18 column (BEH C18 2.1 mm × 100 mm, 1.7 µm particle size, Waters, Guyancourt, France). The flow rate was set as 450 µL/min. The injection volume was 5 µL and the eluents were 0.1% formic acid in water for A and 0.1% formic acid in acetonitrile for B. The eluent B varied as follows: 0 min, 25%; 0.5 min, 25%; 3.6 min, 50%; 3.9 min, 50%; 4 min, 100%; 5.7 min, 100%; 5.8 min, 25%; 7 min, 25%. The column and sample temperature were 30 °C and 20 °C, respectively. The Exactive benchtrop Orbitrap mass spectrometer equipped with a HESI probe (both from Thermo Fisher Scientific, Bremen, Germany) was used. The source parameters were optimized as follows: sheath gas flow rate 65 arbitrary units (a.u.); auxiliary gas flow rate 15 a.u.; sweep gas flow rate 3 a.u.; spray voltage −3.5 kV; capillary temperature 350 °C; capillary voltage −60 V; tube lens voltage −135 V; skimmer voltage −26 V and HESI probe temperature 320 °C. Full MS scan data were acquired in negative ion mode within the range of *m*/*z* 100–1800 at a resolution of 25,000 FWHM. The automatic gain control target was set at 1e6 ions, with a maximum injection time of 100 ms. Data generated from XCalibur (version 2.1, Thermo Fisher Scientific, Waltham, MA, USA) with Qualbrowser and Quanbrowser, and area under curves (AUC) were determined by integrating peaks obtained from extracted ion chromatograms built in a 5-ppm window around the theoretical mass of the deprotonated ions [M-H]- (at *m*/*z* 453.1343 u and *m*/*z* 629.1664 for εVin and εVG, respectively).

### 2.5. Method Validation

The method validation was carried out in accordance with the United States Food and Drug Administration [52] and the European Medicines Agency Guidelines [53] as previously described in other pharmacokinetic studies [54,55,56,57,58,59].

#### 2.5.1. Calibration Curves and Limit of Quantification (LOQ)

The LOQ of εVin and V2G were determined by injecting each concentration 5 times and calculating the relative standard deviation (RSD). LOQs were defined as the lowest concentrations for which trueness (deviation from nominal value) and precision (RSD) were less than 15%. The calibration curve of each compound was determined by integrating peak areas from appropriated extracted ion chromatogram. Each calibration curve was done by spiking the analytes in extracted blank plasma. The correlation coefficient (R²) ensured good linearity (>0.9).

#### 2.5.2. Selectivity, Carry-Over, Intra-Day Precision and Accuracy

Each sample was realized in triplicate. Blank plasmas spiked with εVin or V2G at the highest concentrations were directly injected before blank solvent to perform a carry-over test. To ensure a good selectivity, blank plasmas were also injected after solvent spiked with εVin or V2G. For both tests, peaks at the same retention time of compounds were integrated. An area under curve (AUC) of non-spiked blank plasma and blank solvent inferior to 20% of LOQ ensured an acceptable selectivity and carry-over, respectively.

An intra-day test was performed by spiking low concentration (2.5 ng/mL for εVin and 25 ng/mL for V2G, LC), medium concentration (25 ng/mL for εVin and 250 ng/mL for V2G, MC) and high concentration (250 ng/mL for εVin and 2500 ng/mL for V2G, HC) in solvent (methanol:water, 50:50, *v*:*v*) and in five replicates. RSD (%) and relative error (RE, %) from the nominal value were calculated to determine precision and accuracy, respectively.

#### 2.5.3. Matrix Effect (ME) and Extraction Recovery (ER)

LC, MC and HC were spiked in extracted blank plasma in five replicates. Each concentration was also prepared in solvent (methanol:water, 50:50, *v*:*v*) in five replicates. After integrating each peak in the extracted ion chromatogram, ME was calculated as follows:

$$\mathrm{ME}=\frac{\mathrm{AUC}\;\mathrm{analyte}\;\mathrm{spiked}\;\mathrm{in}\;\mathrm{blank}\;\mathrm{plasma}\;\mathrm{after}\;\mathrm{extraction}}{\mathrm{AUC}\;\mathrm{analyte}\;\mathrm{in}\;\mathrm{solvent}}*100$$

For ER, blank plasmas were spiked with 4 µL of three concentrations prepared in ultra-pure water before operating the extraction protocol. Concentrations were calculated in order to obtain final concentration of LC, MC and HC after extractions, each in five replicates. Fifteen other samples were prepared spiking with the same concentrations in solvent (three concentrations, 5 replicates). After integrating each peak in the extracted ion chromatogram, ER was calculated as follows:

$$\mathrm{ER}=\frac{\mathrm{AUC}\;\mathrm{analyte}\;\mathrm{spiked}\;\mathrm{in}\;\mathrm{blank}\;\mathrm{plasma}\;\mathrm{before}\;\mathrm{extraction}}{\mathrm{AUC}\;\mathrm{analyte}\;\mathrm{in}\;\mathrm{solvent}}*100$$

### 2.6. Data Analysis

Mean concentration-time profiles of εVin and εVG in the rats at scheduled sampling times were analyzed with the non-compartmental pharmacokinetic method using PKSolver (an Excel add-on), and maximum concentration (C_max_) and time to reach C_max_ (T_max_) were recorded directly. Significant differences of εVin and εVG concentrations in tissues and plasmas were determined using a Student’s *t*-test.

## 3. Results

### 3.1. εVin-Loaded MLL Characterization

The encapsulation of εVin was carried out by following the optimized protocol developed previously [48]. The multi-lamellar character was checked by polarized light microscopy. Indeed, the organized structure of MLLs induces birefringence that produces Maltese crosses, as previously observed [48]. Their average size was 247 ± 8 nm as measured by DLS, while their Zeta potential was −47.0 ± 0.4 mV, in accordance with previous results [45,48]. The encapsulation efficiency was 75 ± 5%. After separating the free εVin from the encapsulated one by ultra-centrifugation (see Section 2.2.1), the pellets were analyzed in UPLC-MS to administer a precise quantity of εVin to the animals.

### 3.2. Validation of Extraction and LC-HRMS Quantitation Method

The extraction and the analytical method for the quantification of εVin and V2G in plasma were validated using five replicates in terms of sensitivity, selectivity, carry-over, intraday precision, accuracy, matrix effect and extraction recovery.

The correlation coefficient of the calibration curves (R² = 0.999 for εVin and V2G) ensured a good linearity. LOQ was determined at 0.5 ng/mL for εVin and 25 ng/mL for V2G. No significant peak was measured at the retention times of the compounds, which ensured acceptable selectivity (data not shown). Blank plasmas injected after plasmas spiked with HC did not contain εVin or its glucuronide forms, which validated the carry-over test (data not shown). For the intraday precision and accuracy tests, RSD (%) and RE (%) were calculated respectively, from blank plasmas spiked with LC, MC or HC (*n* = 5). For the three tested concentrations, both precision and accuracy were less than 15% for εVin, and only RE was higher than 15% for V2G in low concentrations (Table 1).

Results for matrix effect and extraction recovery are shown in Table 2. For εVin, the matrix effect was considered negligible. On the other hand, we can note a slight decrease in peak intensity for the extraction recovery measurement. For V2G, there was no impact of the extraction process on metabolite signal but a significant matrix effect with a signal loss of about 30%. The establishment of the standard curves directly in the matrix allows us to free ourselves from this ME.

### 3.3. Pharmacokinetic Study

**Figure 2:**
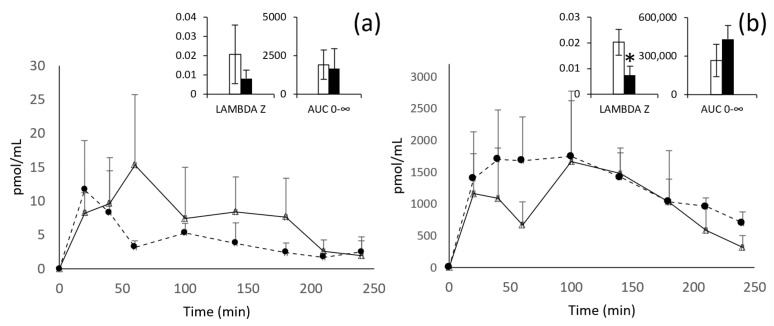
Kinetic profiles of εVin (**a**) and εVG (**b**) after oral administration of 20 mg/kg bw of free (white triangles) or encapsulated (black circles) εVin. Data are expressed in pmol/mL of plasma + standard deviation (SD). Lambda z and AUC_0–∞_ are expressed in min^−1^ and pmol/mL/min, respectively. Comparisons between free (white) or encapsulated (black) forms administration were analyzed by Student’s *t*-test (* *p* < 0.05).

The evolution of the plasma concentrations over time of εVin (expressed in pmol/mL of εVin) and the total glucuronide metabolites (expressed in pmol/mL of V2G, the main metabolite) are shown in Figure 2. The main pharmacokinetic parameters are detailed in the inserts of Figure 2 and in Table 3.

After oral administration of the free form of εVin 20 mg/kg bw, the plasma concentration of the untransformed form reached its maximum concentration after one hour (C_max_ = 15 ± 11 pmol/mL). The total glucuronides reached their C_max_ after 100 min and have a concentration 108 times higher than the aglycone form. For these glucuronides, two peaks of concentration are clearly visible at 20 and 100 min (Figure 2b). 

Considering the encapsulation of εVin in MLLs, despite apparent difference in the plasma kinetic profiles, no significant effect was observed in all the pharmacokinetic parameters followed for the original form (εVin). However, after administration of the encapsulated form of εVin, glucuronides are also the predominant form present in plasma. Moreover, encapsulation of εVin modifies the kinetics of εVG. Indeed, a plateau is observed from 20 min to 100 min, instead of the two peaks obtained with free εVin. Moreover, the time required for the elimination of 50% (T_1/2_) of the glucuronide metabolites is significantly increased, nearly 3 times (from 38 ± 11 min to 119 ± 65, *p* < 0.05) by the encapsulation procedure, in accordance with a significant decrease of the late elimination constant (Lambda z) in the same range of magnitude (from 0.0198 ± 0.0056 min^−1^ to 0.0074 ± 0.0036 min^−1^, *p* < 0.05) and a tendency to increase in AUC_0–∞_ (Table 3, Figure 2b insert).

### 3.4. Quantification in Tissue

**Figure 3:**
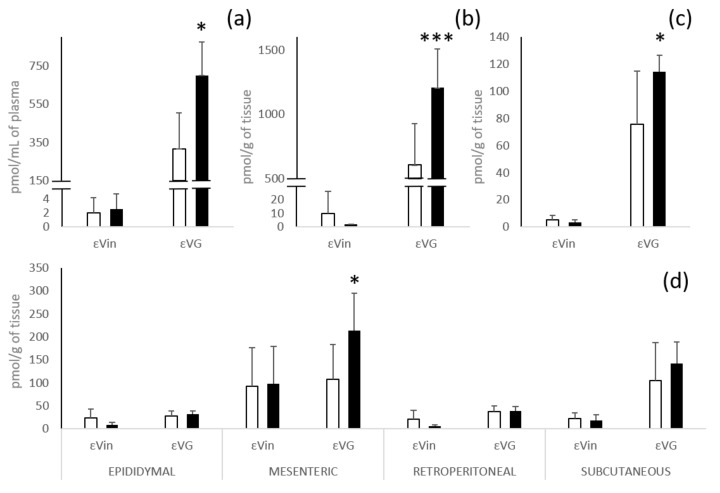
Quantification of εVin and εVG in plasma (**a**); liver (**b**); kidneys (**c**); and different white adipose tissues (**d**) 4 h after oral administration of 20 mg/kg bw of εVin in free (white bars) or encapsulated (black bars) forms. Bars represent concentrations expressed at pmol/mL for plasma and pmol/g of tissue for tissue + SD. Comparisons between encapsulated or free form administration were analyzed by Student’s *t*-test (* *p* < 0.05; *** *p* < 0.0005).

The εVin biodistribution displays great differences in terms of concentration but also of native/glucuronide form ratio according to the tissues (Figure 3).

After administration of the free form of εVin, total glucuronides are the majority forms in plasma, liver, and kidneys. In liver and kidneys, εVin does not exceed a concentration of 10 pmol/g, while εVG reached 604 ± 320 and 76 ± 39 pmol/g of tissue, respectively (60 times and 14 times the concentration of the native molecule) (Figure 3b,c). These marked higher contents of εVG in plasma, liver and kidneys are not observed in adipose tissues (Figure 3d). The highest concentration of the native form was found in mesenteric adipose tissue (92 ± 84 pmol/g of tissue versus concentrations around 20 pmol/g of tissue for the three other depots). For the glucuronides form, only subcutaneous adipose tissue had a much higher εVG concentration than εVin (105 ± 83 versus 23 ± 12 pmol/g of tissue). Four hours after administration, the concentrations of εVin in all the adipose tissues are always largely higher (5 to 10 times) than those measured in the other tissues (liver and kidneys) and plasmas.

The administration of the same amount of εVin, in an encapsulated form, significantly increased (nearly double) the concentration of εVG in plasma (317 ± 186 to 700 ± 175 pmol/mL), liver (604 ± 320 to 1206 ± 304 pmol/g of tissue), kidneys (76 ± 39 to 114 ± 12 pmol/g of tissue) and also in the mesenteric adipose tissue (108 ± 75 pmol/g to 213 ± 81 pmol/g of tissue) (Figure 3). This effect was not observed for the native form.

Independently of the administration form of εVin, it was observed that the levels of εVin and εVG were markedly higher (2 to 3 times) in mesenteric adipose tissue than in the three other adipose tissues, except for the level of εVG in the subcutaneous depot. Finally, it looked like encapsulation led to a slight decrease in the concentration of native form after administration of MLLs in epididymal and retroperitoneal tissues.

## 4. Discussion

This study was carried out with the aim to specifically quantify a resveratrol dimer, ε-viniferin and its main metabolites, namely the mono-glucuronide forms, in biological samples after oral administration of this stilbene with or without an encapsulation procedure.

*Trans*-ε-viniferin-glucuronide production allowed its specific quantification in rat plasmas and tissues. In this study, we used one isomer of glucuronide where the glucuronide moiety is linked to the thirteenth carbon of the molecule (V2G, Figure 1). V2G, which is the major isomer found in vitro after incubation with hepatic fractions [50] and in vivo after intraperitoneal injection of εVin in rat [33], is also the main glucuronides isomers formed of this study for both administration forms. Indeed, after oral administration, V2G is largely in majority followed by V3G, and some traces of V1G and V4G (data not shown). Sulfated metabolites, in low amounts in the in vivo study after intraperitoneal administration [33], were not found in this study.

In the only study on the bioavailability of εVin in rodents, the authors measured its pharmacokinetic parameters, without measuring those of the metabolites. Authors used an oral administration of 40 mg/kg bw, which is two times higher than the amount we used in our study (20 mg/kg bw). They obtained a C_max_ and an AUC_0-∞,_ 2.8 and 3.6 times higher than our values, respectively. All these results confirm that εVin is absorbed after oral administration and that its plasma concentration increases with the quantity administrated [31]. The pharmacokinetic analysis of free εVin in plasma revealed a strong glucuronidation of εVin after oral administration of 20 mg/kg bw of the native form. Indeed, the maximal concentration of glucuronide forms is more than 100 times higher than the concentration of native form. After intraperitoneal administration of 50 mg/kg bw of εVin, Courtois et al. demonstrated that glucuronides were only 1.2 times more concentrated in plasma than the native form [33]. Although in the latter study εVG was expressed in εVin equivalents, underestimating the glucuronide concentration by about 8-fold, it is shown here that εVin undergoes much greater glucuronidation when administered orally than intraperitoneally. This could be explained by an intense intestinal metabolism of εVin, as it has been already demonstrated for resveratrol [60,61]. Concerning these glucuronides, the two peaks on the kinetic of their plasma concentrations, at 20 and 100 min, could match with an enterohepatic recirculation of these metabolites. This enterohepatic cycle has already been demonstrated for glucuronic metabolites of other polyphenols, such as quercetin [62], but never for metabolites of stilbenes to our knowledge. However, different studies on stilbenes have shown that the second absorption peak of the native form occurs at different times depending on the molecule, the doses and the animal model used. This second absorption peak appeared around 60 min after oral administration of pterostilbene [63] and resveratrol [64,65] or more than 10 h for the trimer, α-viniferine [66], for example. Supplemental experiments such as bile analysis could confirm this hypothesis and determine in what form the molecule undergoes this enterohepatic circulation [67,68].

The impact of εVin encapsulation in MLLs on plasma εVG concentrations (appearance of a plateau) could be explained by a sustained release of εVin from the MLLs in the lumen of the digestive tract. Indeed, the MLLs could be gradually degraded during the different digestion stages and more particularly in the lumen of the digestive tract, thus increasing the duration of exposure of εVin to the intestinal wall. Very few studies reported the use of encapsulated active compounds with MLLs for an oral administration in vivo in rats or other laboratory animals. However, Freund et al. (2001) reported that, after oral administration, encapsulation of ^111^In-NTA (labeled amino-polycarboxylic acid) in spherulites led to a greater stability of the compound into the different gastrointestinal levels compared to the free form of the same compound [69]. Moreover, it was reported that MLLs’ encapsulation of ^111^In-NTA significantly increases its plasma concentration in rats [70]. These authors suggested that the encapsulation process is able to increase the bioavailability of compound. In our study, encapsulation does not modify the plasma concentrations of the native molecule (εVin) but shows a tendency to increase that of the metabolites (εVG).

In addition, the Lambda-z, i.e., the terminal elimination rate constant, is significantly smaller for εVG when εVin has been administrated under its encapsulated form, which is correlated with the increase in the T_1/2_ of the compound in plasma. The significant variations of these two parameters, a trend of increase in the AUC_0–∞_, as well as the significant increase of εVG concentrations in plasma, liver and kidneys at 4 h suggest a slower elimination and thus a longer exposure time of εVG in the organism. The use of the encapsulated form could therefore result in a better and prolonged biological efficacy of the glucuronide compounds. Indeed, it is suggested today that glucuronide compounds have biological properties [71]; for example, it has recently been shown that resveratrol and pterostilbene are able to reduce steatosis in cultured hepatocytes [72,73].

According to tissue analysis, εVin was found in higher concentrations in white adipose tissues than in plasma, liver and kidneys 4 h after oral administration. This important presence of the native form in white adipose tissues had already been demonstrated after intraperitoneal administration [33]. However, this is the first time that it has been shown that, although it is extensively metabolized, a stilbene is stored in relatively large quantities in its native form in white adipose tissues after oral administration. The native form being more lipophilic than the glucuronide ones might suggest that they are stored in the unilocular lipid droplets in adipocytes. Adipose tissues could thus act as a reservoir for εVin after having captured the native circulating form, or after the cleavage of the glucuronide group of the metabolites. Unlike the intraperitoneal study, the glucuronide forms are also found in adipose tissues, testifying to the strong intestinal metabolism after oral administration.

Interestingly, the εVin and εVG contents were higher in the mesenteric depot than in the three other ones (except for εVG in the subcutaneous one), the encapsulation of εVin significantly increasing the accumulation of the glucuronide metabolites in this adipose tissue. Mesenteric adipose tissue, dispersed throughout the small intestine, is highly vascularized with a great metabolic activity in the post-prandial state [74]. Thus, if these anatomical and functional differences could logically explain the increased amount of εVin and εVG, we also do not rule out possible plasma contamination of the tissues during their sampling. However, the hypothesis of proximity to the digestive system or of contamination with the plasma cannot justify the relatively high concentrations found in the subcutaneous fatty tissues, again suggesting a storage role of adipose tissues.

## 5. Conclusions

Our study showed significant glucuronidation of εVin which occurred mainly in the liver and probably also in the intestine after oral administration. The presence of εVin and its glucuronide metabolites in adipose tissues was also demonstrated, which means that adipose tissues could represent a storage tissue, but also be a target tissue in which the dimer could regulate lipid metabolism.

This study also showed that, although encapsulation did not improve the plasma concentrations of εVin, its main impact was to prolong the exposure of organism to its glucuronide metabolites. Therefore, now that the encapsulation protocol has been developed, it could be used to study the promising biological application of εVin for human health.

## Figures and Tables

**Table 1:** Intraday precision of εVin and V2G in rat plasma (*n* = 5).

	Level	Spiked Concentration (ng/mL)	Mean Concentration (ng/mL)	RSD (%)	RE (%)
εVin	LC	2.5	2.5 ± 0.2	9.1	0.8
	MC	25	23.4 ± 0.4	1.6	−6.4
	HC	250	249.6 ± 4.6	1.8	−0.2
V2G	LC	25	33.9 ± 0.7	2.1	35.6
	MC	250	236.1 ± 3.3	1.4	−5.5
	HC	2500	2504.9 ± 45.1	1.8	0.2

**Table 2:** Matrix effect and extraction recovery of εVin and V2G in rat plasma.

			Matrix Effect		Extraction Recovery	
	Level	Spiked Concentration (ng/mL)	Mean ± SD (%)	RSD (%)	Mean ± SD (%)	RSD (%)
εVin	LC	2.5	93.3 ± 7.2	7.8	87.2 ± 30.1	34.5
	MC	25	115.1 ± 2.3	2.0	87.2 ± 3.8	4.4
	HC	250	103.7 ± 4.4	4.2	74.7 ± 1.4	7.8
V2G	LC	25	70.5 ± 15.2	21.5	114.7 ± 14.4	12.6
	MC	250	69.2 ± 4.5	6.5	96.2 ± 7.8	8.1
	HC	2500	69.5 ± 4.2	6.1	96.8 ± 4.2	4.3

**Table 3:** Pharmacokinetic parameters of εVin and εVG in rat plasma after oral administration of free or encapsulated form. T_max_, T_1/2_ and mean residence time (MRT) were expressed in minutes, C_max_ in pmol/mL, AUC in pmol/mL/min and Lambda z in min^−1^. Comparisons between encapsulated or free form administration were analyzed by Student’s *t*-test (* *p* < 0.05).

	εVin		εVG	
	Free	Encapsulated	Free	Encapsulated
T_max_	60	20	100	100
C_max_	15.3 ± 10.5	11.7 ± 7.3	1667.4 ± 1103.3	1746.3 ± 875.6
T_½_	55.0 ± 33.9	114.8 ± 80.4	38.2 ± 11.2	118.8 ± 65.1 *
AUC_0__-__t_	1689.1 ± 682.0	1009.8 ± 328.3	245,827.3 ± 114,219.8	307,086.4 ± 316,383.9
AUC_0–∞_	1919.7 ± 955.2	1660.1 ± 1313.1	264,191.0 ± 123,786.4	432,315.4 ± 106,406.4
MRT	126.9 ± 27.9	196.4 ± 130.4	123.7 ± 12.0	213.2774 ± 93.9
Lambda z	0.0207 ± 0.0153	0.0082 ± 0.0043	0.0198 ± 0.0056	0.0074 ± 0.0036 *

## Data Availability

The data presented in this study are available upon request from the corresponding author.
